# The seeds of *Plantago lanceolata* comprise a stable core microbiome along a plant richness gradient

**DOI:** 10.1186/s40793-024-00552-x

**Published:** 2024-02-02

**Authors:** Yuri Pinheiro Alves de Souza, Michael Schloter, Wolfgang Weisser, Yuanyuan Huang, Stefanie Schulz

**Affiliations:** 1 TUM School of Life Science, Chair of Environmental Microbiology, Technische Universität München, Neuherberg, Germany; 2 Research Unit Comparative Microbiome Analysis, Helmholtz Zentrum München, Neuherberg, Germany; 3 TUM School of Life Science, Chair of Terrestrial Ecology, Technische Universität München, Freising, Germany; 4 German Centre of Integrative Bioaffiliationersity Research (iDiv) Halle-Jena-Leipzig, Leipzig, Germany; 5 Institute of Biology, Experimental Interaction Ecology, Leipzig University, Leipzig, Germany

**Keywords:** *Plantago lanceolata*, Seed microbiome, Plant diversity gradient, Core microbiome

## Abstract

**Background:**

Seed endophytic bacteria are beneficial to plants. They improve seedling growth by enhancing plant nutrient uptake, modulating stress-related phytohormone production, and targeting pests and pathogens with antibiotics. Seed endophyte composition can be influenced by pollination, plant cultivar, and soil physicochemical conditions. However, the effects of plant community richness on seed endophytes are unknown. To investigate the effects of increasing plant species richness on the diversity and composition of the seed microbiome, we made use of a well-established long-term biodiversity experiment in Germany (The Jena Experiment). We sampled seeds from different *Plantago lanceolata* blossoms in a plant diversity gradient ranging from monoculture to 16 species mixtures. The seeds were surface sterilized to remove seed surface-associated bacteria and subjected to a metabarcoding approach to assess bacterial community structure.

**Results:**

Our data indicate a very stable core microbiome, which accounted for more than 90% of the reads and was present in all seeds independent of the plant richness level the seeds originated from. It consisted mainly of reads linked to *Pseudomonas rhizosphaerae, Sphingomonas faeni* and *Pirellulla spp*. 9% of the obtained reads were not part of the core microbiome and were only present in plots of specific diversity levels. The number of unique ASVs was positively correlated with plant richness. Interestingly, most reads described as non-core members belonged to the same genera described as the core microbiome, indicating the presence of different strains or species with possibly different functional properties important for seed performance.

**Conclusion:**

Our data indicate that *Plantago lanceolata* maintains a large seeds core microbiome across the plant richness gradient. However, the number of unique ASVs increases alongside the plant community richness, indicating that ecosystem biodiversity also mitigates diversity loss in seed endophytes.

**Supplementary Information:**

The online version contains supplementary material available at 10.1186/s40793-024-00552-x.

## Background

Seed endophytic microbial communities play an essential role in seed protection and germination [1, 2]. Moreover, the seed microbiome contributes to important responses during seedling development and in early plant life stages, acting as the initial inoculum for the microbiome of the next plant generation and representing a heritage link between the parental plant and its offspring [3]. Changes in the structure and function of the endophytic seed microbiome can critically affect the development of the seedling by modulating growth as well as stress responses of the plant at early stages of life [3]. Especially for domesticated plants a reduction in seed microbiome diversity was observed with negative consequences for plant resistance against pathogen invasion, for example [4].

A study by Bergna et al. [5] indicates that the seed microbiome composition is determined by parental filtering, which selects microbes from the air, water and soil. On the one hand, this filtering results in plant species specific and even genotype specific seed microbiomes as it was shown for rice [6], *Cucurbita pepo* [7] or oilseed rape [8]. On the other hand, environmental conditions modulate the composition of the seed microbiome as well as it was shown for the type and presence of pollinators [9] or differences in soil properties [10]. However, the effect of increasing plant diversity has not been explored so far. Although many studies proved the positive effect of diverse plant communities on several ecosystem functions [11–13]. A similar positive link between plant diversity and seed endophytic microbial diversity can indicate that ecosystem biodiversity can impact not only the endophytic microbiome of the current plant generation but also their offspring, representing an important legacy effect of biodiversity increase [10].

Here, we hypothesize that plant diversity also influences the structure and diversity of the seed bacteria. Therefore, we used *Plantago lanceolata*, a plant that is widely distributed in European grasslands and is often used as a model in ecological studies [12, 14]. We analyzed the seed microbiome of *P. lanceolata* in frame of the setting provided by “The Jena Experiment” [12, 14], one of the largest plant diversity experiments worldwide, and enabled us to compare changes in the seed microbiome in monocultures, 4-, 8- and 16-species plant communities.

## Methods

### Experimental design and sampling

This experiment was conducted in the frame of “The Jena Experiment” [6, 7 - http://the-jena-experiment.de/], which is running since 2002. The experiment was established in the city of Jena, Germany, next to the Saale River (50°55’43.61"N, 11°35’23.64"E). A pool of 60 regional grassland species was combined to create a diversity gradient, which resulted in 82 plots (20 m × 20 m; 400 m²). The gradient spans from monoculture plots to 60-species mixtures. For our experiment, we sampled only plots that had *Plantago lanceolata* in their mixture. Thus, seeds from all available monoculture plots (3) and from 4-, 8- and 16-species mixtures were sampled, which were 4, 2 and 3 plots, respectively. Detailed information about plant taxonomical composition of each sampled plot as well as the identity of the direct neighbor of the sampled *P. lanceolata* individuals can be found in Supplementary Table S1. The *P. lanceolata* coverage in the 60-species plots was too low for additional sampling. This summed up to 12 plots, which were sampled in September 2021. The sampling was performed in a nested design, which is depicted in Supplementary Figure S1. Three individual plants were sampled per plot, and three blossoms were sampled per individual. Blossoms were collected using gloves and surface-disinfected scissors. Intact blossoms were stored in sterile 15 ml falcon tubes and transported to the laboratory at room temperature where the seeds were removed from the blossoms. The resulting seeds were surface sterilized as follows: 1 min incubation in 1% Tween; 2 min incubation in 70% ethanol; 3 washes in sterile water; 5 min incubation in 5% sodium hypochlorite solution; and 3 washes in sterile water [15]. The surface-sterilized seeds were stored at -20 °C until further processing.

### DNA extraction

Surface-sterilized seeds were ground using sterilized mortars and liquid nitrogen. Per DNA extraction, 50 seeds were used, equaling 97.5 mg of seeds. The number of seeds was estimated based on the weight of 1000 *P. lanceolata* seeds, which is 1.95 g for plants from “The Jena Experiment” field site [16]. For blossoms with a lower amount of seeds, all material was used. Seed DNA was extracted following a phenol/chloroform/isoamyl alcohol-based method [17]. Sample lysis was performed using Lysing Matrix E tubes (MP Biomedicals™, Germany). The beat beating was done using a TissueLyser II bead beater (QIAGEN®, Germany) at a frequency of 15 Hz for 2 min. The resulting DNA was quantified by Qubit fluorometric system (Thermo Fisher Scientific, Germany) using the broad range assay kit. The quality of the DNA was checked using the Nanodrop photometric system (Thermo Fisher Scientific, Germany) and by agarose gel electrophoresis. To exclude contamination during DNA extraction, a blank control without seed material was processed.

### Amplicon library preparation and sequencing

We performed metabarcoding targeting the V3 and V4 regions of the 16S rRNA gene using chloroplast exclusion primers S-D-Bact-0335-a-S-17 (338f -TCGTCGGCAGCGTCAGATGTGTATAAGAGACAGCADACTCCTACGGGAGGC) and S-D-Bact-0769-a-A-19 (789r-GTCTCGTGGGCTCGGAGATGTGTATAAGAGACAGATCCTGTTTGMTMCCCVCRC) [18] with overhang sequences at the 5’ end compatible with the Nextera® XT Index Kit. The primers used reduce the overamplification of plant derived 16 S rRNA genes including chloroplast and mitochondria sequences, while targeting the same region of the 16 S rRNA gene as the frequently used earth microbiome primers [19, 20] which improves comparability of data sets across different habitats. PCR amplification was performed using 20 ng of template DNA, and negative controls without DNA template were processed alongside. Each PCR consisted of 25 µL containing 12.5 µL NEB Next High-Fidelity Master Mix (Thermo Fisher Scientific, Germany), 0.5 µL of each primer at 10 pmol/µl, 2.5 µL of 3% BSA, 1 µl of 5 ng/µL diluted DNA, and 8 µL of DEPC-treated water. The thermal profile was as follows: 98 °C for 1 min, followed by 35 cycles of 98 °C for 10 s, 55 °C for 30 s and 72 °C for 30 s, and a final extension at 72 °C for 5 min, which was repeated 35 times. Samples were indexed using the Nextera® XT Index Kit v2 (Illumina, USA) and purified with MagSi-NGSprep Plus Beads (ratio 0.8 beads:1 sample) according to the manufacturer’s protocol, and quality assessment was performed via the Fragment Analyzer System 5300 (Agilent, Germany). High-quality DNA was diluted to 4 nM and sequenced on an Illumina MiSeq using a MiSeq Reagent v3 (600 Cycle) kit. PhiX (5 pM, 20%) was loaded alongside the samples. Raw sequencing files were uploaded to the NCBI SRA database under the bio project number PRJNA937585 and bio sample SAMN33409010.

### Sequence processing

After sequencing, samples were uploaded to the European Galaxy server (https://usegalaxy.eu). The Cutadpat [21] tool was used to remove adapters, and the quality of reads was assessed via FastQC [22]. Forward reads with quality scores below 30 and reverse reads with quality scores below 20 were removed. For further analysis, dada2 version 1.16 [23] was used. The plotQualityProfile option was used to determine the trimming parameters, which were set to 280 bp for the forward reads and 220 bp for the reverse reads. The next steps included the calculation of error rates and sample inference followed by merging reads and removal of chimeric sequences (https://benjjneb.github.io/dada2/tutorial.html). The loss of reads during that process is summarized in Table S1. Taxonomy was assigned using assignTaxonomy and addSpecies function, aligning the amplicon sequence variants (ASVs) against the Silva database [24] version 138.

### Statistical analyses

Plots and statistical analysis were conducted in R version 4.2.2 using the packages phyloseq version 1.42.0 [25] and vegan [26] version 4.0.5. Before analysis, all ASVs detected in both extraction and PCR negativecontrols were removed from the dataset. This resulted in 8.772 reads distributed among 129 ASVs. Additionally, reads taxonomically assigned to chloroplast or mitochondria were removed. The sample specific read loss during sample processing is summarized in Table S2. In total, 4007 different ASVs were detected in the full dataset. To estimate whether the sequencing depth of the remaining reads was enough to reach sufficient coverage, rarefaction curves were drawn using the *rarecurve* command in the package Vegan v 2.6.4. Nine samples with fewer than 1000 reads were excluded from further downstream analysis. Afterwards, the blossoms per individual were treated as ps pseudo-replicates per plant individual level. Thus, only consensus sequences detected on all three blossoms per individual were further considered. For the statistical analyses, plant species richness (SR) was treated as continuous variable, and it was log2-transformed to reach linearity. To estimate alpha diversity, the number of observed ASVs was used as a richness estimate and calculated using the *estimate_richness* command in Phyloseq. We fitted the transformed data to a linear mixed-effects model using the lme function of package *lme4* [27] to investigate the effects of plant species richness on the number of observed ASVs (AD). We applied random term corrections to the plot (PL) and individual (IN) levels. The fitting order was m1<-lme(AD ~ log2(SR), random = ~ 1|PL/IN). Beta diversity was calculated by using the Bray‒Curtis dissimilarity matrix as input for a PCoA using the *ordinate* command from phyloseq [25]. Significant differences in community composition were tested using PERMANOVA (*p* < 0.05). To account for the dependency of samples coming from the same plot we used the *strata* command in the adonis2 package [28] to restrict permutations within the plot (999 permutations). In addition to the sown plant richness gradient, we calculated plant realized diversity by using species-level aboveground plant biomass data from August 2021 [29, 30] to calculate both the Shannon and Simpson diversity indices. Biomass data was obtained by harvesting all aboveground plant materials using a 0.1 m² (20 × 50 cm) frame randomly positioned in each plot. The Shannon and Simpson indices were calculated using the “diversity” function from the vegan package (version 2.6-4). We used the calculated plant diversity to calculate the correlation between the number of observed bacterial ASVs per plot to the actual realized plant diversity in each plot. The core microbiome was calculated using the *trans_venn* function of the package MicroEco [31] v 0.14.2. Venn diagrams were drawn taking into consideration 100% prevalence, meaning that only ASVs present in all individuals of a given plant richness level were considered.

## Results

On average, we obtained 19,625 high-quality reads per sample (Table S2) and reached sufficient coverage of the bacterial richness in the seeds of *P. lanceolata* for all samples, as depicted in Supplementary Figure S2 (Additional file 1).

**Fig. 1:**
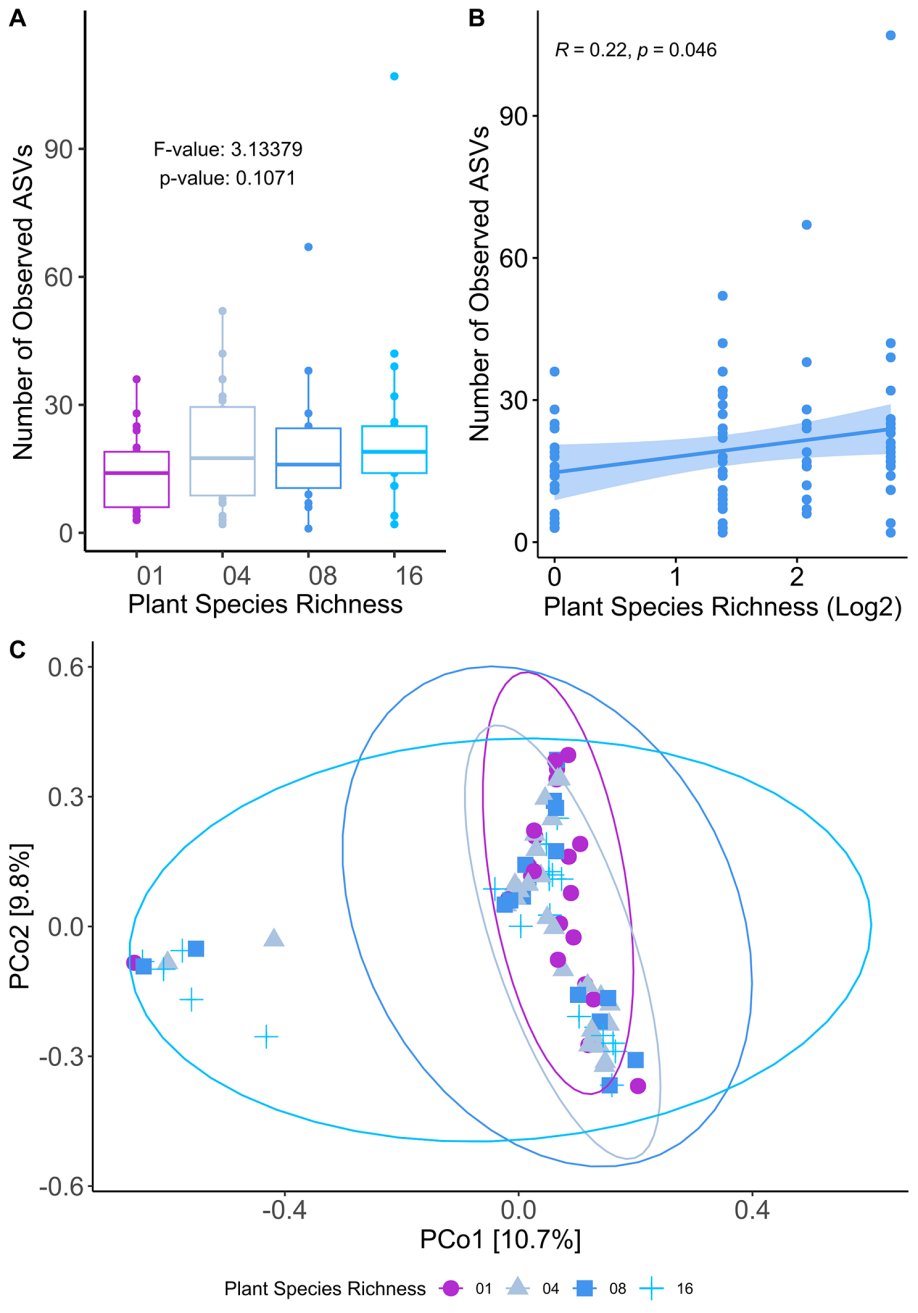
**(A)** Number of observed ASVs per plant richness level and linear mixed effects models used to investigate the relationship between plant richness and the number of bacterial observed ASVs. A nested design was applied where Plot and Individual were used as random term in the model, correcting for grouping effects. **(B)** Spearman correlation plot between the number of observed ASVs and plant species richness (log2 scale) **(C)** PcoA analyzes built over Bray–Curtis dissimilarity distance

**Fig. 2:**
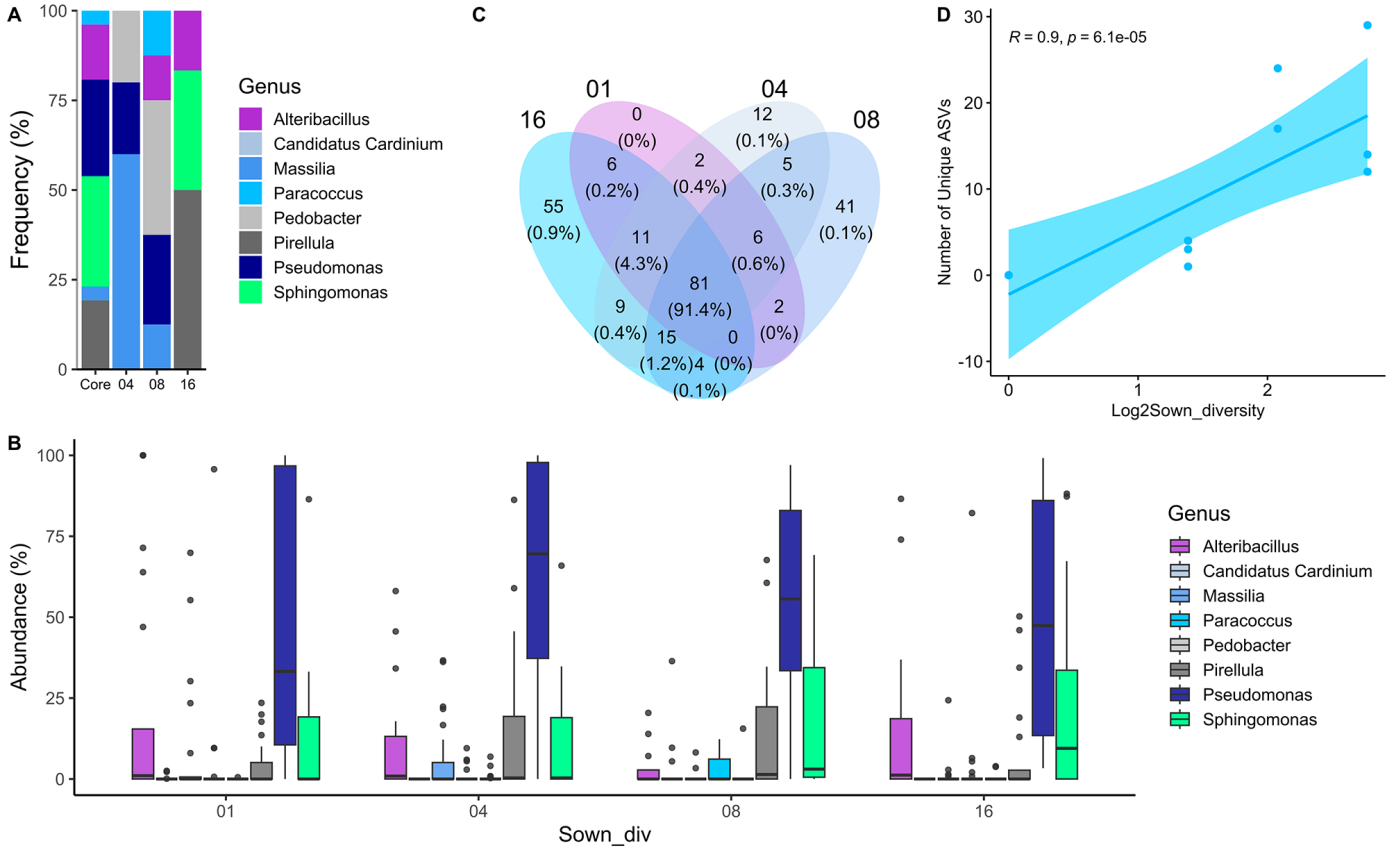
The seed microbiome of *P. lanceolata* across the different plant diversity levels. **(A)** Frequency and phylogenetic assignment of ASVs belonging to the core microbiome (Core) or the unique taxa of 4-, 8- and 16-species plots. Monoculture plots did not harbor any unique ASV. **(B)** Relative abundance of ASVs being part of the core microbiome in seeds obtained from plants of plots from the different diversity levels (Sown_div). **(C)** Venn diagram displaying the number of shared ASVs among the diversity levels. Numbers outside brackets indicate the absolute number of ASVs shared on that given combination, while numbers inside brackets represent the percentage of reads assigned to those ASVs. Relative abundances below 0.1% are displayed as 0%. **(D)** Spearman correlation plot between the number of unique ASVs per diversity level and plant species diversity displayed as log2sown diversity. In monocultures, the number of unique ASVs is zero

In line with no significant effects of plant richness on overall seed microbiome diversity (lme; F: 3.13379, *p*: 0.1071 Fig. 1A ), our analysis identified a stable core microbiome of *P. lanceolata* seeds across the plant diversity gradient. The core microbiome contained 91% of all detected reads, explaining the poor separation of samples when ß-diversity was analyzed (PCoA on Bray–Curtis dissimilarity; PERMANOVA: *p* = 0.401– Fig. 1C). We observed, however, a slight positive correlation between the number of observed ASVs and the plant richness gradient (*R* = 0.22, *p* = 0.046 - Fig. 1B), however no correlation was observed when the number of observed ASVs was correlated with realized Shannon and Simpson plant diversity on each plot (*R* = 0.085, *p* = 0.44 for Shannon realized diversity and *R* = 0.14, *p* = 0.21 - Supplementary Figure S3, Additional file 1). The core microbiome consisted of 81 ASVs assigned to the genera *Paracoccus*, *Alteribacillus*, *Sphingomonas*, *Pseudomonas*, *Massilia* and *Pirellula* (Fig. 2A). Among those, *Pseudomonas rhizosphaerae* followed by *Sphingomonas faeni* and *Pirellulla spp.* were the most abundant species of the core microbiome (Fig. 2B).

In contrast to the core microbiome, the number of unique ASVs at each plant richness level was positively correlated with plant richness (*R* = 0.9, *p* = 0.00006, Fig. 2C and D). The identity of unique ASVs of the *P. lanceolata* seed microbiome differed at the different plant richness levels and mostly consisted of rare ASVs. Interestingly, no unique ASVs were found in the seeds of the monoculture plots, while 12 unique ASVs could be detected in seeds from 4 species plots, which further increased to 41 and 55 unique ASVs in samples from 8 to 16 plant species plots, respectively (Fig. 2C). Interestingly, most of the ASVs detected in the core microbiome and among the unique ASVs belonged to the same genera.

## Discussion

In the present study, we demonstrate for the first time that the microbiome of *Plantago lanceolata* seeds is highly conserved across the tested plant richness gradient. However, we also observed that the stable core microbiome is extended by additional unique ASVs, whose number was positively correlated with plant richness.

Previous studies reported that seed endophytic bacteria are present in extremely low counts, especially in surface-sterilized seeds [4, 32]. We acknowledged these low biomass properties during our data analysis in two ways. First, we processed blank extraction as well as PCR negative controls and removed all ASVs found in those controls from the samples. Second, to account for the high oscillation in low biomass samples [33, 34], we worked with the consensus community per plant individual, which was achieved by conglomerating the sequencing results of three individual blossoms (biological replicates) per plant as explained in the methods section. This procedure allowed the removal of 90% of the ASVs, which were not consistent within the same individual. Thus, we are confident that the obtained results are robust and do not originate from contamination or random fluctuation.

As result of our study, we identified a large, conserved bacterial core microbiome of *P. lanceolata* seeds across the plant richness gradient. This indicates that *P. lanceolata* is capable of recruiting a very specific set of bacteria from the environmental pool, even though the surrounding plant richness and community composition was not the same. On the one hand, this finding is in line with other studies, which identified large seed core microbiomes of similar plant species. For example, Eyre et al. (2019) [6] described a consistent core microbiome for rice coming from different geographical locations and genotypes. However, rice and other crops are mostly cultivated in monocultures and based on breeding efforts which drive comparable traits, namely productivity. On the other hand, previous studies demonstrated that increasing plant richness significantly changed ecosystem functions and thus the properties of the surrounding habitat. For example, the number and diversity of pollinators increases with plant richness and the soil microbial community composition changes [12]. Both directly change the sources from which the seed microbiome is selected from. However, it might be possible that these effects are bigger for the microbiome colonizing the seed surface [6], which was excluded in our analysis. Most of the detected bacterial taxa have already been described as part of the seed microbiome of other plant species. For example, *Pseudomonas* and *Alternaria* were identified as consistent seed endophytes in a metastudy, which compared 50 different plant species [2]. *Pseudomonas rhizosphaerae* has been previously isolated from vegetal tissue, demonstrating important plant growth-promoting traits such as phosphate solubilization [35], while *Sphingomonas faeni*, originally isolated from indoor dust, belongs to a genus widely known for its phytohormone production [36]. To date, *Pirelulla* has been mostly found in marine samples and has not yet been described in plant tissues [37].

In addition to the core microbiome, the number of unique ASVs of the *P. lanceolata* seed microbiome increased with plant richness, but their identity differed at the different plant richness levels. We attribute the increasing number of additional ASVs to changes in parental filtering processes [10]. Current literature suggests that microbial recruitment of seed endophytes depends on plant topology and phytochemistry [38]. Studies on the plant properties along the investigated plant richness gradient revealed that plant phenotypes, genotypes and chemical defense mechanisms [12, 39, 40] significantly differed in *P. lanceolata* monocultures compared to plots with higher plant richness. Thus, the recruitment of additional taxa might be related to changes in plant performance along the plant richness gradient. These differences seemed to induce a stricter selection in plots with low plant richness, which are characterized by a higher pathogen load for example [12]. Similar trends were observed for crop monocultures with long term domestication history [4].The increasing number of unique ASVs at plant species rich plots provides additional complexity of the endophytic seed bacteria along the plant richness gradient. The beneficial effects of increasing diversity on ecosystem stability have been vastly investigated [12, 41, 42]. Biodiversity enhances the stability and resilience of ecosystems by improving the complementary use of resources and functional redundancy [43], which prevents ecosystems to collapse during disturbances [16, 44]. Although the positive feedback of plant richness on the diversity of soil [45] and rhizosphere microbiomes has already been observed [45, 46], this interaction has not been observed for the bacterial seed endophytes. The data indicates that the beneficial effects of mixed plantations can be carried on to the next generation to a certain extent, possibly improving seed defense, seedling germination and overall plant robustness [1, 47].

Interestingly, most of the ASVs detected in the core microbiome and among the unique ASVs belonged to the same genera, indicating a differentiation between core and unique taxa below the genus level. Differences in the manifestation of plant growth promotion traits have been reported at the species or strain level [48], indicating the importance of the non-core ASVs for the plant performance of the next generation. Only ASVs linked to the genus *Pedobacter* were exclusively found in plots with 4 and 8 plant species, apparently being sorted out by parental filtering on the 16 plant species level plot. *Pedobacter* has been described as widely ubiquitous in soil and water [49] and was recently described as a plant endophyte of *Carex pumilia* [50]. Interestingly, *Carex* and *Plantago* are only poorly phylogenetically related but have been described as functionally similar in grasslands [51], which might also be linked to the previously undescribed functional profiles of *Pedobacter.* The strong influence of functional traits on the selection of seed endophytes was also observed for hyperaccumulating plants, which conserved specific plant growth promoting bacteria across different plant families [52].

## Conclusion

Our study identified a stable seed core microbiome of *P. lanceolata* along a plant richness gradient. The core taxa include bacteria frequently identified as plant growth promoting bacteria underlining the important role of the transgenerational transfer of endophytic bacteria to ensure the provision of a consistent starter community for the next plant generation. In addition to the maintenance of a specific core microbiome, our data suggests that this can be supplemented by additional taxa if *P. lanceolata* is grown in plant species rich grasslands. The number of these additional taxa was positively correlated with the number of plant species on the plot. This indicates that the beneficial effects of mixed plantations can be carried on to the next generation, possibly improving seed defense, seedling germination and overall plant robustness [1, 47]. Future studies should investigate whether a similar pattern can be found for other plant species and other microbial groups like fungi or protists to figure out if this might be a future strategy to mitigate consequences of domestication. Moreover, the investigation of consequences for the performance of the next plant generation are open questions resulting from our study.

### Electronic supplementary material

Below is the link to the electronic supplementary material.

Supplementary Material 1

## Data Availability

The dataset supporting the conclusions of this article is included within the article (and its Additional file 1). The datasets generated and/or analyzed during the current study are available in the NCBI SRA database repository under project number PRJNA937585, biosample SAMN33409010. R script and metadata files can be found at https://github.com/Streptomyces1/plantago_seed_microbiome.
