# Supplementary material for: The seeds of Plantago lanceolata comprise a stable core microbiome along a plant richness gradient
Source: Environ Microbiome. 2024 Feb 2;19:11. doi: 10.1186/s40793-024-00552-x (PMC10835927; doi:10.1186/s40793-024-00552-x)
Supplement: Supplementary file 1 — Supplementary Material 1 [file 40793_2024_552_MOESM1_ESM.docx]

# **Supplementary Material**

The seeds of *Plantago lanceolata* comprise a stable core microbiome along a plant richness gradient

Yuri Pinheiro ^1, 2^; Michael Schloter ^1, 2^; Wolfgang Weisser ^3^; Yuanyuan Huang^4,5^, Stefanie Schulz ^1*^

^1^Technische Universität München, TUM School of Life Science, Chair of Environmental Microbiology, Freising, Germany

^2^Helmholtz Zentrum München, Research Unit Comparative Microbiome Analysis, Neuherberg, Germany

^3^ Technische Universität München, TUM School of Life Science, Chair of Terrestrial Ecology, Freising, Germany

^4^German Centre of Integrative Biodiversity Research (iDiv) Halle-Jena-Leipzig, Germany

^5^Institute of Biology, Experimental Interaction Ecology, Leipzig University, Germany

*corresponding author: [schloter@tum.de](mailto:schloter@tum.de)


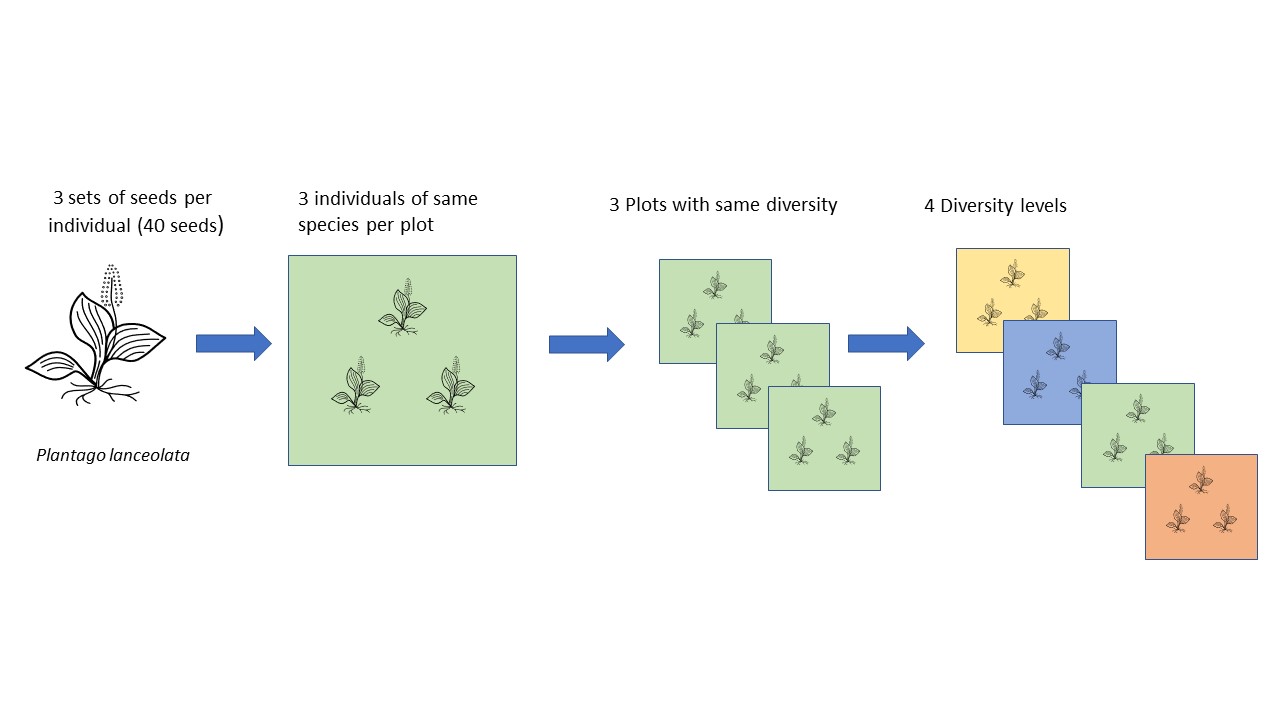


Supplementary Figure S1: Seed sampling scheme. Blossoms were samples on each diversity level containing Plantago lanceolata in the Jena experiment. In each of the 12 sampled plots, 3 individuals were sampled, each individual is composed by 3 blossoms.


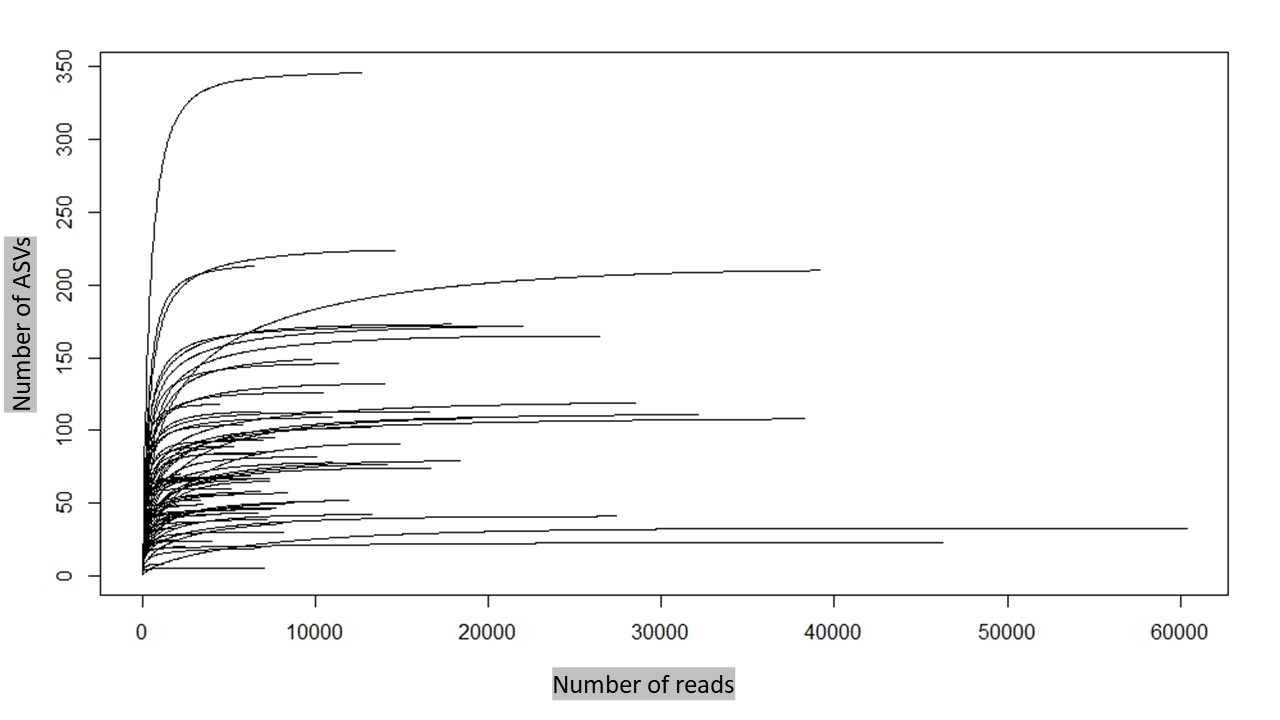


Supplementary Figure S2: Rarefaction curve drawn using the ASV table prevenient from metabarcoding sequencing.

Table S1: Metadata information with block, plot, indivudal, replicate code, neighbor information of each sampled individual and taxonomical compostion of each sampled plot.

| **Sample** | **Code** | **Block** | **Plot** | **Replicate** | **Plant Species Richness** | **Individual** | **Neighbor_1** | **Neighbor_2** | **Neighbor_3** | | | **Plot_Species** | |
| --- | --- | --- | --- | --- | --- | --- | --- | --- | --- | --- | --- | --- | --- |
| 1_S72 | B1A01-1-1 | B1 | B1A01 | B1A01-1-1 | 16 | A | *Knautia arvensis* | *Plantago lanceolata* | *Galium mollugo* | | | *Anthoxanthum odoratum; Avenula pubescens; Bromus erectus; Poa pratensis; Ajuga reptans; Plantago lanceolata; Ranunculus repens; Taraxacum officinale; Anthriscus sylvestris; Carum carvi; Geranium pratense; Tragopogon pratensis; Lathyrus pratensis; Lotus corniculatus; Trifolium campestre; Vicia cracca* | |
| 2_S73 | B1A01-1-2 | B1 | B1A01 | B1A01-1-2 | 16 | A | *Knautia arvensis* | *Plantago lanceolata* | *Galium mollugo* | | |  |  |
| 3_S74 | B1A01-1-3 | B1 | B1A01 | B1A01-1-3 | 16 | A | *Knautia arvensis* | *Plantago lanceolata* | *Galium mollugo* | | |  |  |
| 4_S75 | B1A01-2-1 | B1 | B1A01 | B1A01-2-1 | 16 | B | *Ranunculus repens* | *Geranium pratense* |  | | |  |  |
| 5_S76 | B1A01-2-2 | B1 | B1A01 | B1A01-2-2 | 16 | B | *Ranunculus repens* | *Geranium pratense* |  | | |  |  |
| 6_S77 | B1A01-2-3 | B1 | B1A01 | B1A01-2-3 | 16 | B | *Ranunculus repens* | *Geranium pratense* |  | | |  |  |
| 7_S78 | B1A01-3-1 | B1 | B1A01 | B1A01-3-1 | 16 | C | *Geranium pratense* | *Plantago lanceolata* | *Galium mollugo* | | |  |  |
| 8_S79 | B1A01-3-2 | B1 | B1A01 | B1A01-3-2 | 16 | C | *Geranium pratense* | *Plantago lanceolata* | *Galium mollugo* | | |  |  |
| 9_S80 | B1A01-3-3 | B1 | B1A01 | B1A01-3-3 | 16 | C | *Geranium pratense* | *Plantago lanceolata* | *Galium mollugo* | | |  |  |
| 13_S81 | B1A04-1-1 | B1 | B1A04 | B1A04-1-1 | 4 | A | *Arenaterum elatius* | *Picris hieracioides* |  | | | *Festuca pratensis; Plantago lanceolata; Campanula patula; Onobrychnis viciifolia* | |
| 14_S82 | B1A04-1-2 | B1 | B1A04 | B1A04-1-2 | 4 | A | *Arenaterum elatius* | *Picris hieracioides* |  | | |  |  |
| 15_S83 | B1A04-1-3 | B1 | B1A04 | B1A04-1-3 | 4 | A | *Arenaterum elatius* | *Picris hieracioides* |  | | |  |  |
| 16_S84 | B1A04-2-1 | B1 | B1A04 | B1A04-2-1 | 4 | B | *Arenaterum elatius* | *Picris hieracioides* |  | | |  |  |
| 17_S85 | B1A04-2-2 | B1 | B1A04 | B1A04-2-2 | 4 | B | *Arenaterum elatius* | *Picris hieracioides* |  | | |  |  |
| 18_S86 | B1A04-2-3 | B1 | B1A04 | B1A04-2-3 | 4 | B | *Arenaterum elatius* | *Picris hieracioides* |  | | |  |  |
| 19_S87 | B1A04-3-1 | B1 | B1A04 | B1A04-3-1 | 4 | C | *Arenaterum elatius* | *Picris hieracioides* |  | | |  |  |
| 20_S88 | B1A04-3-2 | B1 | B1A04 | B1A04-3-2 | 4 | C | *Arenaterum elatius* | *Picris hieracioides* |  | | |  |  |
| 21_S89 | B1A04-3-3 | B1 | B1A04 | B1A04-3-3 | 4 | C | *Arenaterum elatius* | *Picris hieracioides* |  | | |  |  |
| 25_S90 | B1A14-1-1 | B1 | B1A14 | B1A14-1-1 | 8 | A | *Knautia arvensis* | *Plantago lanceolata* | *unidentified grass* | | | *Luzula campestris; Trisetum flavescens; Leontodon hispidus; Plantago lanceolata; Anthriscus sylvestris; Daucus carota; Trifolium campestre; Trifolium fragiferum* | |
| 26_S91 | B1A14-1-2 | B1 | B1A14 | B1A14-1-2 | 8 | A | *Knautia arvensis* | *Plantago lanceolata* | *unidentified grass* | | |  |  |
| 27_S92 | B1A14-1-3 | B1 | B1A14 | B1A14-1-3 | 8 | A | *Knautia arvensis* | *Plantago lanceolata* | *unidentified grass* | | |  |  |
| 28_S93 | B1A14-2-1 | B1 | B1A14 | B1A14-2-1 | 8 | B | *unidentified grass* | *Knautia arvensis* |  | | |  |  |
| 29_S94 | B1A14-2-2 | B1 | B1A14 | B1A14-2-2 | 8 | B | *unidentified grass* | *Knautia arvensis* |  | | |  |  |
| 30_S95 | B1A14-2-3 | B1 | B1A14 | B1A14-2-3 | 8 | B | *unidentified grass* | *Knautia arvensis* |  | | |  |  |
| 31_S96 | B1A14-3-1 | B1 | B1A14 | B1A14-3-1 | 8 | C | *Trifolium pratense* | *Plantago lanceolata* | *Knautia arvensis* | | |  |  |
| 32_S97 | B1A14-3-2 | B1 | B1A14 | B1A14-3-2 | 8 | C | *Trifolium pratense* | *Plantago lanceolata* | *Knautia arvensis* | | |  |  |
| 33_S98 | B1A14-3-3 | B1 | B1A14 | B1A14-3-3 | 8 | C | *Trifolium pratense* | *Plantago lanceolata* | *Knautia arvensis* | | |  |  |
| 37_S99 | B1A20-1-1 | B1 | B1A20 | B1A20-1-1 | 16 | A | *Knautia arvensis* | *Medicago varia* |  | | | *Ajuga reptans; Bellis perennis; Leontodon autumnalis; Leontodon hispidus; Plantago lanceolata; Veronica chamaedrys; Achillea millefolium; Geranium pratense; Knautia arvensis; Ranunculus acris; Sanguisorba officinalis; Lotus corniculatus; Medicago varia; Onobrychnis viciifolia; Trifolium hybridum; Trifolium repens* | |
| 38_S100 | B1A20-1-2 | B1 | B1A20 | B1A20-1-2 | 16 | A | *Knautia arvensis* | *Medicago varia* |  | | |  |  |
| 39_S101 | B1A20-1-3 | B1 | B1A20 | B1A20-1-3 | 16 | A | *Knautia arvensis* | *Medicago varia* |  | | |  |  |
| 40_S102 | B1A20-2-1 | B1 | B1A20 | B1A20-2-1 | 16 | B | *Trifolium pratense* | *Knautia arvensis* | *Medicago varia* | | |  |  |
| 41_S103 | B1A20-2-2 | B1 | B1A20 | B1A20-2-2 | 16 | B | *Trifolium pratense* | *Knautia arvensis* | *Medicago varia* | | |  |  |
| 42_S104 | B1A20-2-3 | B1 | B1A20 | B1A20-2-3 | 16 | B | *Trifolium pratense* | *Knautia arvensis* | *Medicago varia* | | |  |  |
| 43_S105 | B1A20-3-1 | B1 | B1A20 | B1A20-3-1 | 16 | C | *Geranium pratense* | *Medicago varia* | *Knautia arvensis* | | |  |  |
| 44_S106 | B1A20-3-2 | B1 | B1A20 | B1A20-3-2 | 16 | C | *Geranium pratense* | *Medicago varia* | *Knautia arvensis* | | |  |  |
| 45_S107 | B1A20-3-3 | B1 | B1A20 | B1A20-3-3 | 16 | C | *Geranium pratense* | *Medicago varia* | *Knautia arvensis* | | |  |  |
| 49_S108 | B1B045-1-1 | B1 | B1B045 | B1B045-1-1 | 1 | A | *Plantago lanceolata* |  |  | | *Plantago lanceolata (monoculture)* | |  |
| 50_S109 | B1B045-1-2 | B1 | B1B045 | B1B045-1-2 | 1 | A | *Plantago lanceolata* |  |  | |  |  |  |
| 51_S110 | B1B045-1-3 | B1 | B1B045 | B1B045-1-3 | 1 | A | *Plantago lanceolata* |  |  | |  |  |  |
| 52_S111 | B1B045-2-1 | B1 | B1B045 | B1B045-2-1 | 1 | B | *Plantago lanceolata* |  |  | |  |  |  |
| 53_S1 | B1B045-2-2 | B1 | B1B045 | B1B045-2-2 | 1 | B | *Plantago lanceolata* |  |  | |  |  |  |
| 54_S2 | B1B045-2-3 | B1 | B1B045 | B1B045-2-3 | 1 | B | *Plantago lanceolata* |  |  | |  |  |  |
| 58_S3 | B1B045-4-1 | B1 | B1B045 | B1B045-4-1 | 1 | C | *Plantago lanceolata* |  |  | |  |  |  |
| 59_S4 | B1B045-4-2 | B1 | B1B045 | B1B045-4-2 | 1 | C | *Plantago lanceolata* |  |  | |  |  |  |
| 60_S5 | B1B045-4-3 | B1 | B1B045 | B1B045-4-3 | 1 | C | *Plantago lanceolata* |  |  | |  |  |  |
| 61_S6 | B1C076-1-1 | B1 | B1C076 | B1C076-1-1 | 1 | A | *Plantago lanceolata* |  |  | | *Plantago lanceolata (monoculture)* | |  |
| 62_S7 | B1C076-1-2 | B1 | B1C076 | B1C076-1-2 | 1 | A | *Plantago lanceolata* |  |  | |  |  |  |
| 63_S8 | B1C076-1-3 | B1 | B1C076 | B1C076-1-3 | 1 | A | *Plantago lanceolata* |  |  | |  |  |  |
| 67_S9 | B1C076-3-1 | B1 | B1C076 | B1C076-3-1 | 1 | B | *Plantago lanceolata* |  |  | |  |  |  |
| 68_S10 | B1C076-3-2 | B1 | B1C076 | B1C076-3-2 | 1 | B | *Plantago lanceolata* |  |  | |  |  |  |
| 69_S11 | B1C076-3-3 | B1 | B1C076 | B1C076-3-3 | 1 | B | *Plantago lanceolata* |  |  | |  |  |  |
| 70_S12 | B1C076-4-1 | B1 | B1C076 | B1C076-4-1 | 1 | C | *Plantago lanceolata* |  |  | |  |  |  |
| 71_S13 | B1C076-4-2 | B1 | B1C076 | B1C076-4-2 | 1 | C | *Plantago lanceolata* |  |  | |  |  |  |
| 72_S14 | B1C076-4-3 | B1 | B1C076 | B1C076-4-3 | 1 | C | *Plantago lanceolata* |  |  | |  |  |  |
| 76_S15 | B2A06-2-1 | B2 | B2A06 | B2A06-2-1 | 4 | A | *Cirsium arvense* |  |  | | | *Plantago lanceolata; Taraxacum officinale; Lotus corniculatus; Medicago lupulina* | |
| 77_S16 | B2A06-2-2 | B2 | B2A06 | B2A06-2-2 | 4 | A | *Cirsium arvense* |  |  | | |  |  |
| 78_S17 | B2A06-2-3 | B2 | B2A06 | B2A06-2-3 | 4 | A | *Cirsium arvense* |  |  | | |  |  |
| 79_S18 | B2A06-3-1 | B2 | B2A06 | B2A06-3-1 | 4 | B | *Cirsium arvense* |  |  | | |  |  |
| 80_S19 | B2A06-3-2 | B2 | B2A06 | B2A06-3-2 | 4 | B | *Cirsium arvense* |  |  | | |  |  |
| 81_S20 | B2A06-3-3 | B2 | B2A06 | B2A06-3-3 | 4 | B | *Cirsium arvense* |  |  | | |  |  |
| 83_S22 | B2A06-4-2 | B2 | B2A06 | B2A06-4-2 | 4 | C | *Bromus erectus* |  |  | | |  |  |
| 84_S23 | B2A06-4-3 | B2 | B2A06 | B2A06-4-3 | 4 | C | *Bromus erectus* |  |  | | |  |  |
| 85_S24 | B2A09-1-1 | B2 | B2A09 | B2A09-1-1 | 4 | A | *Trifolium pratense* | *Prunella vulgaris* | *Knautia arvensis* | | | *Ajuga reptans; Plantago lanceolata; Primula veris; Prunella vulgaris* | |
| 86_S25 | B2A09-1-2 | B2 | B2A09 | B2A09-1-2 | 4 | A | *Trifolium pratense* | *Prunella vulgaris* | *Knautia arvensis* | | |  |  |
| 87_S26 | B2A09-1-3 | B2 | B2A09 | B2A09-1-3 | 4 | A | *Trifolium pratense* | *Prunella vulgaris* | *Knautia arvensis* | | |  |  |
| 91_S27 | B2A09-3-1 | B2 | B2A09 | B2A09-3-1 | 4 | B | *Prunella vulgaris* | *Primula veris* |  | | |  |  |
| 92_S28 | B2A09-3-2 | B2 | B2A09 | B2A09-3-2 | 4 | B | *Prunella vulgaris* | *Primula veris* |  | | |  |  |
| 93_S70 | B2A09-3-3 | B2 | B2A09 | B2A09-3-3 | 4 | B | *Prunella vulgaris* | *Primula veris* |  | | |  |  |
| 94_S29 | B2A09-4-1 | B2 | B2A09 | B2A09-4-1 | 4 | C | *Primula veris* | *Prunella vulgaris* |  | | |  |  |
| 95_S30 | B2A09-4-2 | B2 | B2A09 | B2A09-4-2 | 4 | C | *Primula veris* | *Prunella vulgaris* |  | | |  |  |
| 96_S31 | B2A09-4-3 | B2 | B2A09 | B2A09-4-3 | 4 | C | *Primula veris* | *Prunella vulgaris* |  | | |  |  |
| 98_S32 | B2A10-1-2 | B2 | B2A10 | B2A10-1-2 | 16 | A | *unidentified grass* | *Geranium pratense* | *Primula veris* | | | *Alopecurus pratensis; Anthoxanthum odoratum; Arrhenaterum elatius; Bromus erectus; Festuca pratensis; Holcus lanatus; Phleum pratense; Poa pratensis; Bellis perennis; Leontodon autumnalis; Leontodon hispidus; Plantago lanceolata; Primula veris; Prunella vulgaris; Ranunculus repens; Veronica chamaedrys* | |
| 99_S33 | B2A10-1-3 | B2 | B2A10 | B2A10-1-3 | 16 | A | *unidentified grass* | *Geranium pratense* | *Primula veris* | | |  |  |
| 100_S34 | B2A10-2-1 | B2 | B2A10 | B2A10-2-1 | 16 | B | *unidentified grass* | *Geranium pratense* | *Primula veris* | | |  |  |
| 101_S35 | B2A10-2-2 | B2 | B2A10 | B2A10-2-2 | 16 | B | *unidentified grass* | *Primula veris* |  | | |  |  |
| 102_S36 | B2A10-2-3 | B2 | B2A10 | B2A10-2-3 | 16 | B | *unidentified grass* | *Primula veris* |  | | |  |  |
| 103_S37 | B2A10-3-1 | B2 | B2A10 | B2A10-3-1 | 16 | C | *unidentified grass* | *Primula veris* |  | | |  |  |
| 104_S38 | B2A10-3-2 | B2 | B2A10 | B2A10-3-2 | 16 | C | *unidentified grass* |  |  | | |  |  |
| 105_S39 | B2A10-3-3 | B2 | B2A10 | B2A10-3-3 | 16 | C | *unidentified grass* |  |  | | |  |  |
| 109_S40 | B2A13-1-1 | B2 | B2A13 | B2A13-1-1 | 1 | A | *Plantago lanceolata* | | |  | | *Plantago lanceolata (monoculture)* | |
| 110_S41 | B2A13-1-2 | B2 | B2A13 | B2A13-1-2 | 1 | A | *Plantago lanceolata* | | |  | |  |  |
| 111_S42 | B2A13-1-3 | B2 | B2A13 | B2A13-1-3 | 1 | A | *Plantago lanceolata* | | |  | |  |  |
| 115_S43 | B2A13-3-1 | B2 | B2A13 | B2A13-3-1 | 1 | B | *Equisetum arvense* | *Plantago lanceolata* |  | | |  | |
| 116_S44 | B2A13-3-2 | B2 | B2A13 | B2A13-3-2 | 1 | B | *Equisetum arvense* | *Plantago lanceolata* |  | | |  |  |
| 117_S45 | B2A13-3-3 | B2 | B2A13 | B2A13-3-3 | 1 | B | *Equisetum arvense* | *Plantago lanceolata* |  | | |  |  |
| 118_S46 | B2A13-4-1 | B2 | B2A13 | B2A13-4-1 | 1 | C | *Campanula barbara* | *Plantago lanceolata* |  | | |  |  |
| 119_S47 | B2A13-4-2 | B2 | B2A13 | B2A13-4-2 | 1 | C | *Campanula barbara* | *Plantago lanceolata* |  | | |  |  |
| 120_S48 | B2A13-4-3 | B2 | B2A13 | B2A13-4-3 | 1 | C | *Campanula barbara* | *Plantago lanceolata* |  | | |  |  |
| 124_S49 | B4A08-2-1 | B4 | B4A08 | B4A08-2-1 | 8 | A | *unidentified grass* | *Crepis vesicaria* |  | | | *Avenula pubescens; Bromus hordeaceus; Festuca rubra; Ajuga reptans; Plantago lanceolata; Taraxacum officinale; Veronica chamaedrys; Anthoxanthum odoratum* | |
| 125_S50 | B4A08-2-2 | B4 | B4A08 | B4A08-2-2 | 8 | A | *unidentified grass* | *Crepis vesicaria* |  | | |  |  |
| 126_S51 | B4A08-2-3 | B4 | B4A08 | B4A08-2-3 | 8 | A | *unidentified grass* | *Crepis vesicaria* |  | | |  |  |
| 127_S52 | B4A08-3-1 | B4 | B4A08 | B4A08-3-1 | 8 | B | *Picris hieracioides* | *Plantago lanceolata* | *unidentified grass* | | |  |  |
| 128_S53 | B4A08-3-2 | B4 | B4A08 | B4A08-3-2 | 8 | B | *Picris hieracioides* | *Plantago lanceolata* | *unidentified grass* | | |  |  |
| 129_S54 | B4A08-3-3 | B4 | B4A08 | B4A08-3-3 | 8 | B | *Picris hieracioides* | *Plantago lanceolata* | *unidentified grass* | | |  |  |
| 130_S55 | B4A08-4-1 | B4 | B4A08 | B4A08-4-1 | 8 | C | *Picris hieracioides* |  |  | | |  |  |
| 131_S56 | B4A08-4-2 | B4 | B4A08 | B4A08-4-2 | 8 | C | *Picris hieracioides* |  |  | | |  |  |
| 136_S58 | B4A04-2-1 | B4 | B4A04 | B4A04-2-1 | 4 | A | *Picris hieracioides* |  |  | | | *Arrhenaterum elatius; Plantago lanceolata; Anthriscus sylvestris; Trifolium dubium* | |
| 137_S59 | B4A04-2-2 | B4 | B4A04 | B4A04-2-2 | 4 | A | *Arenaterum elatius* | *Picris hieracioides* |  | | |  |  |
| 138_S60 | B4A04-2-3 | B4 | B4A04 | B4A04-2-3 | 4 | A | *Arenaterum elatius* | *Picris hieracioides* |  | | |  |  |
| 139_S61 | B4A04-3-1 | B4 | B4A04 | B4A04-3-1 | 4 | B | *Arenaterum elatius* | *Picris hieracioides* |  | | |  |  |
| 140_S62 | B4A04-3-2 | B4 | B4A04 | B4A04-3-2 | 4 | B | *Arenaterum elatius* | *Picris hieracioides* |  | | |  |  |
| 141_S63 | B4A04-3-3 | B4 | B4A04 | B4A04-3-3 | 4 | B | *Arenaterum elatius* | *Picris hieracioides* |  | | |  |  |
| 142_S64 | B4A04-4-1 | B4 | B4A04 | B4A04-4-1 | 4 | C | *Arenaterum elatius* | *Picris hieracioides* |  | | |  |  |
| 143_S65 | B4A04-4-2 | B4 | B4A04 | B4A04-4-2 | 4 | C | *Arenaterum elatius* | *Picris hieracioides* |  | | |  |  |
| 144_S66 | B4A04-4-3 | B4 | B4A04 | B4A04-4-3 | 4 | C | *Arenaterum elatius* | *Picris hieracioides* |  | | |  |  |

Table S2: Table displaying the read loss per processing step during the bioinformatic pipeline. Samples with blank in their name display extraction controls and with NTC PCR controls.

| **Sample** | **Individual** | **filter+trim reads.in** | **filter+trim reads.out** | **dadaF** | **dadaR** | **merged** | **seqtable** | **removeBimera** | **Removal chloroplast and mitochondria** |
| --- | --- | --- | --- | --- | --- | --- | --- | --- | --- |
| 1_S72 | B1A01-1 | 13050 | 10625 | 10574 | 10453 | 10320 | 10320 | 10262 | 3774 |
| 100_S34 | B1A01-1 | 15141 | 10594 | 10415 | 10413 | 9968 | 9968 | 9706 | 1846 |
| 101_S35 | B1A01-1 | 53412 | 46343 | 45770 | 45874 | 44007 | 44007 | 35860 | 14083 |
| 102_S36 | B1A01-2 | 15071 | 12098 | 11921 | 11940 | 11103 | 11103 | 10444 | 3288 |
| 103_S37 | B1A01-2 | 63345 | 55796 | 55126 | 55170 | 50905 | 50905 | 38832 | 32087 |
| 104_S38 | B1A01-2 | 19135 | 16770 | 16504 | 16525 | 15556 | 15556 | 14454 | 7114 |
| 105_S39 | B1A01-3 | 73279 | 61862 | 61443 | 61355 | 59331 | 59331 | 48192 | 18910 |
| 109_S40 | B1A01-3 | 40468 | 34990 | 34122 | 34419 | 30368 | 30368 | 24282 | 9687 |
| 110_S41 | B1A01-3 | 9802 | 7033 | 6878 | 6821 | 6278 | 6278 | 6038 | 1302 |
| 111_S42 | B1A04-1 | 28654 | 23512 | 23240 | 23273 | 22239 | 22239 | 21268 | 4762 |
| 115_S43 | B1A04-1 | 16089 | 13732 | 13557 | 13551 | 12971 | 12971 | 11539 | 254 |
| 116_S44 | B1A04-1 | 421 | 367 | 248 | 245 | 214 | 214 | 201 | 3 |
| 117_S45 | B1A04-2 | 84751 | 74984 | 74609 | 74839 | 73491 | 73491 | 47928 | 46257 |
| 118_S46 | B1A04-2 | 964 | 801 | 750 | 735 | 690 | 690 | 666 | 38 |
| 119_S47 | B1A04-2 | 70831 | 60878 | 60304 | 60411 | 57154 | 57154 | 52909 | 13644 |
| 120_S48 | B1A04-3 | 36985 | 30613 | 30198 | 30247 | 28888 | 28888 | 23080 | 8670 |
| 124_S49 | B1A04-3 | 2206 | 1602 | 1540 | 1521 | 1395 | 1395 | 1343 | 162 |
| 125_S50 | B1A04-3 | 6253 | 5485 | 5408 | 5346 | 5155 | 5155 | 4360 | 4032 |
| 126_S51 | B1A14-1 | 9550 | 7993 | 7794 | 7820 | 7235 | 7235 | 5670 | 2410 |
| 127_S52 | B1A14-1 | 62391 | 54566 | 54102 | 54076 | 52250 | 52250 | 42405 | 14838 |
| 128_S53 | B1A14-1 | 9626 | 8423 | 8360 | 8353 | 8261 | 8261 | 8033 | 7043 |
| 129_S54 | B1A14-2 | 73495 | 63980 | 63811 | 63773 | 63382 | 63382 | 62095 | 60218 |
| 13_S81 | B1A14-2 | 85 | 50 | 38 | 32 | 23 | 23 | 23 | 15 |
| 130_S55 | B1A14-2 | 43352 | 36707 | 36174 | 36268 | 34424 | 34424 | 29940 | 2019 |
| 131_S56 | B1A14-3 | 50521 | 43405 | 42993 | 43132 | 41177 | 41177 | 36606 | 6663 |
| 136_S58 | B1A14-3 | 149 | 88 | 38 | 22 | 14 | 14 | 14 | 14 |
| 137_S59 | B1A14-3 | 20697 | 13339 | 12882 | 12783 | 11332 | 11332 | 10940 | 5624 |
| 138_S60 | B1A20-1 | 7725 | 4672 | 4556 | 4538 | 4275 | 4275 | 4002 | 275 |
| 139_S61 | B1A20-1 | 58086 | 48468 | 48167 | 48118 | 46615 | 46615 | 39183 | 27248 |
| 14_S82 | B1A20-1 | 14884 | 12516 | 12207 | 12288 | 10961 | 10961 | 10123 | 5672 |
| 140_S62 | B1A20-2 | 26139 | 20316 | 19904 | 20033 | 18465 | 18465 | 16765 | 9699 |
| 141_S63 | B1A20-2 | 28476 | 24190 | 23854 | 23816 | 22550 | 22550 | 19143 | 7311 |
| 142_S64 | B1A20-2 | 17698 | 13128 | 12909 | 12893 | 11970 | 11970 | 11021 | 2518 |
| 143_S65 | B1A20-3 | 20115 | 16772 | 16553 | 16480 | 15420 | 15420 | 13927 | 8159 |
| 144_S66 | B1A20-3 | 15106 | 13137 | 13037 | 13038 | 12855 | 12855 | 12525 | 6687 |
| 15_S83 | B1A20-3 | 28237 | 23981 | 23433 | 23504 | 20567 | 20567 | 18530 | 12295 |
| 16_S84 | B1B045-1 | 300214 | 260942 | 260129 | 260271 | 253730 | 253730 | 223332 | 113403 |
| 17_S85 | B1B045-1 | 22570 | 17541 | 17193 | 17218 | 15871 | 15871 | 15534 | 7049 |
| 18_S86 | B1B045-1 | 24417 | 20148 | 19740 | 19733 | 17479 | 17479 | 15409 | 11310 |
| 19_S87 | B1B045-2 | 56 | 34 | 16 | 15 | 9 | 9 | 9 | 6 |
| 2_S73 | B1B045-2 | 38404 | 32779 | 32558 | 32550 | 31915 | 31915 | 28940 | 11605 |
| 20_S88 | B1B045-2 | 15704 | 14085 | 13920 | 13897 | 12721 | 12721 | 9561 | 8103 |
| 21_S89 | B1B045-4 | 17316 | 15293 | 15105 | 15159 | 14382 | 14382 | 13523 | 7079 |
| 25_S90 | B1B045-4 | 5850 | 4476 | 4354 | 4283 | 3692 | 3692 | 3587 | 1012 |
| 26_S91 | B1B045-4 | 2173 | 225 | 184 | 178 | 173 | 173 | 173 | 8 |
| 27_S92 | B1C076-1 | 106 | 60 | 38 | 13 | 13 | 13 | 5 | 5 |
| 28_S93 | B1C076-1 | 22302 | 20108 | 19723 | 19643 | 17264 | 17264 | 11542 | 6013 |
| 29_S94 | B1C076-1 | 52147 | 45783 | 45380 | 45406 | 42647 | 42647 | 36651 | 26267 |
| 3_S74 | B1C076-3 | 17363 | 14435 | 14299 | 14158 | 13683 | 13683 | 13395 | 2878 |
| 30_S95 | B1C076-3 | 26 | 14 | 1 | 1 | 0 | 0 | 0 | 0 |
| 31_S96 | B1C076-3 | 18360 | 13291 | 12794 | 12865 | 11267 | 11267 | 10595 | 4810 |
| 32_S97 | B1C076-4 | 24622 | 21588 | 21406 | 21372 | 20757 | 20757 | 19610 | 1565 |
| 33_S98 | B1C076-4 | 21478 | 18837 | 18695 | 18681 | 18112 | 18112 | 17786 | 3322 |
| 37_S99 | B1C076-4 | 18665 | 16450 | 16179 | 16122 | 14255 | 14255 | 13676 | 4448 |
| 38_S100 | B2A06-2 | 33706 | 30263 | 29843 | 29872 | 27681 | 27681 | 19495 | 16690 |
| 39_S101 | B2A06-2 | 27172 | 24139 | 23903 | 23835 | 23054 | 23054 | 18940 | 7687 |
| 4_S75 | B2A06-2 | 16413 | 12130 | 11806 | 11630 | 10487 | 10487 | 9876 | 6154 |
| 40_S102 | B2A06-3 | 30941 | 22359 | 22018 | 22040 | 20172 | 20172 | 19815 | 8850 |
| 41_S103 | B2A06-3 | 46933 | 41687 | 41416 | 41374 | 39533 | 39533 | 37714 | 32323 |
| 42_S104 | B2A06-3 | 8403 | 7389 | 7284 | 7302 | 6722 | 6722 | 5251 | 1916 |
| 43_S105 | B2A06-4 | 25424 | 22105 | 21918 | 21898 | 21572 | 21572 | 21018 | 19902 |
| 44_S106 | B2A06-4 | 33681 | 29605 | 29191 | 29396 | 27315 | 27315 | 24526 | 9194 |
| 45_S107 | B2A09-1 | 17333 | 15752 | 15669 | 15687 | 15432 | 15432 | 14123 | 14026 |
| 49_S108 | B2A09-1 | 7155 | 3939 | 3830 | 3771 | 3435 | 3435 | 3162 | 976 |
| 5_S76 | B2A09-1 | 27080 | 22372 | 22071 | 22052 | 20652 | 20652 | 20066 | 11701 |
| 50_S109 | B2A09-3 | 72233 | 62713 | 62317 | 62375 | 59935 | 59935 | 53240 | 8880 |
| 51_S110 | B2A09-3 | 32309 | 28033 | 27642 | 27717 | 25379 | 25379 | 22146 | 16244 |
| 52_S111 | B2A09-3 | 44476 | 39182 | 38877 | 38961 | 36890 | 36890 | 32047 | 18807 |
| 53_S1 | B2A09-4 | 14678 | 12349 | 12085 | 12101 | 11002 | 11002 | 9486 | 4945 |
| 54_S2 | B2A09-4 | 22610 | 18518 | 18274 | 18356 | 17582 | 17582 | 16731 | 3265 |
| 58_S3 | B2A09-4 | 56298 | 50723 | 50384 | 50383 | 48182 | 48182 | 38624 | 18354 |
| 59_S4 | B2A10-1 | 26448 | 22669 | 22431 | 22471 | 21147 | 21147 | 19480 | 2360 |
| 6_S77 | B2A10-1 | 42606 | 34739 | 34130 | 34183 | 30922 | 30922 | 27912 | 13783 |
| 60_S5 | B2A10-2 | 39415 | 35086 | 34877 | 34925 | 33630 | 33630 | 31536 | 2663 |
| 61_S6 | B2A10-2 | 10404 | 8885 | 8780 | 8725 | 8374 | 8374 | 8176 | 3067 |
| 62_S7 | B2A10-2 | 16998 | 14896 | 14697 | 14673 | 14064 | 14064 | 10327 | 8082 |
| 63_S8 | B2A10-3 | 24583 | 21341 | 21137 | 21076 | 20124 | 20124 | 18561 | 5115 |
| 67_S9 | B2A10-3 | 5771 | 4988 | 4838 | 4845 | 4457 | 4457 | 4154 | 575 |
| 68_S10 | B2A10-3 | 10735 | 9395 | 9297 | 9298 | 9026 | 9026 | 6900 | 6786 |
| 69_S11 | B2A13-1 | 28326 | 24604 | 24422 | 24421 | 23560 | 23560 | 22348 | 2029 |
| 7_S78 | B2A13-1 | 35915 | 30009 | 29588 | 29450 | 26702 | 26702 | 25865 | 15974 |
| 70_S12 | B2A13-1 | 37854 | 33197 | 32957 | 32918 | 31221 | 31221 | 28357 | 6415 |
| 71_S13 | B2A13-3 | 29156 | 25600 | 25406 | 25320 | 24382 | 24382 | 23070 | 3867 |
| 72_S14 | B2A13-3 | 4960 | 4366 | 4296 | 4298 | 4148 | 4148 | 4117 | 1784 |
| 76_S15 | B2A13-3 | 40193 | 34862 | 34209 | 34289 | 29875 | 29875 | 27462 | 22007 |
| 77_S16 | B2A13-4 | 47823 | 41859 | 41373 | 41297 | 37230 | 37230 | 31584 | 25377 |
| 78_S17 | B2A13-4 | 7714 | 6565 | 6403 | 6412 | 5878 | 5878 | 5511 | 650 |
| 79_S18 | B2A13-4 | 21431 | 18924 | 18644 | 18699 | 17263 | 17263 | 14623 | 6250 |
| 8_S79 | B4A08-2 | 12564 | 10658 | 10549 | 10526 | 10033 | 10033 | 9835 | 4602 |
| 80_S19 | B4A08-2 | 32980 | 29650 | 29306 | 29344 | 27359 | 27359 | 23016 | 13194 |
| 81_S20 | B4A08-2 | 12916 | 11353 | 11245 | 11151 | 10598 | 10598 | 9849 | 4113 |
| 82_S21 | B4A08-3 | 11814 | 9977 | 9825 | 9759 | 9402 | 9402 | 8461 | 0 |
| 83_S22 | B4A08-3 | 2973 | 2622 | 2519 | 2472 | 2244 | 2244 | 2168 | 1872 |
| 86_S25 | B4A08-3 | 173822 | 150022 | 148170 | 148895 | 138419 | 138419 | 89751 | 38793 |
| 87_S26 | B4A08-4 | 5489 | 4723 | 4588 | 4627 | 4339 | 4339 | 4226 | 516 |
| 9_S80 | B4A08-4 | 33497 | 29229 | 28739 | 28768 | 25939 | 25939 | 17582 | 10942 |
| 91_S27 | B4A04-2 | 13839 | 11500 | 11398 | 11385 | 10915 | 10915 | 10428 | 1222 |
| 92_S28 | B4A04-2 | 2917 | 2434 | 2372 | 2321 | 2167 | 2167 | 2109 | 1179 |
| 93_S70 | B4A04-2 | 22846 | 18102 | 17288 | 17380 | 14087 | 14087 | 13404 | 12635 |
| 94_S29 | B4A04-3 | 814 | 670 | 638 | 629 | 586 | 586 | 578 | 152 |
| 95_S30 | B4A04-3 | 35151 | 29760 | 29370 | 29493 | 27615 | 27615 | 25326 | 4370 |
| 96_S31 | B4A04-3 | 2040 | 1691 | 1540 | 1529 | 1259 | 1259 | 1212 | 292 |
| 98_S32 | B4A04-4 | 92431 | 81438 | 80701 | 80936 | 76760 | 76760 | 67332 | 37709 |
| 99_S33 | B4A04-4 | 28795 | 25023 | 24730 | 24714 | 23493 | 23493 | 13507 | 13248 |
| Blank_S67 | B4A04-4 | 12305 | 9223 | 9051 | 9087 | 8644 | 8644 | 8063 | REMOVED |
| NTC-1_S68 | NTC | 1192 | 485 | 435 | 411 | 336 | 336 | 329 | REMOVED |
| NTC-2_S69 | NTC | 941 | 680 | 631 | 607 | 535 | 535 | 514 | REMOVED |
| NTC-3_S71 | NTC | 47 | 26 | 11 | 12 | 8 | 8 | 6 | REMOVED |


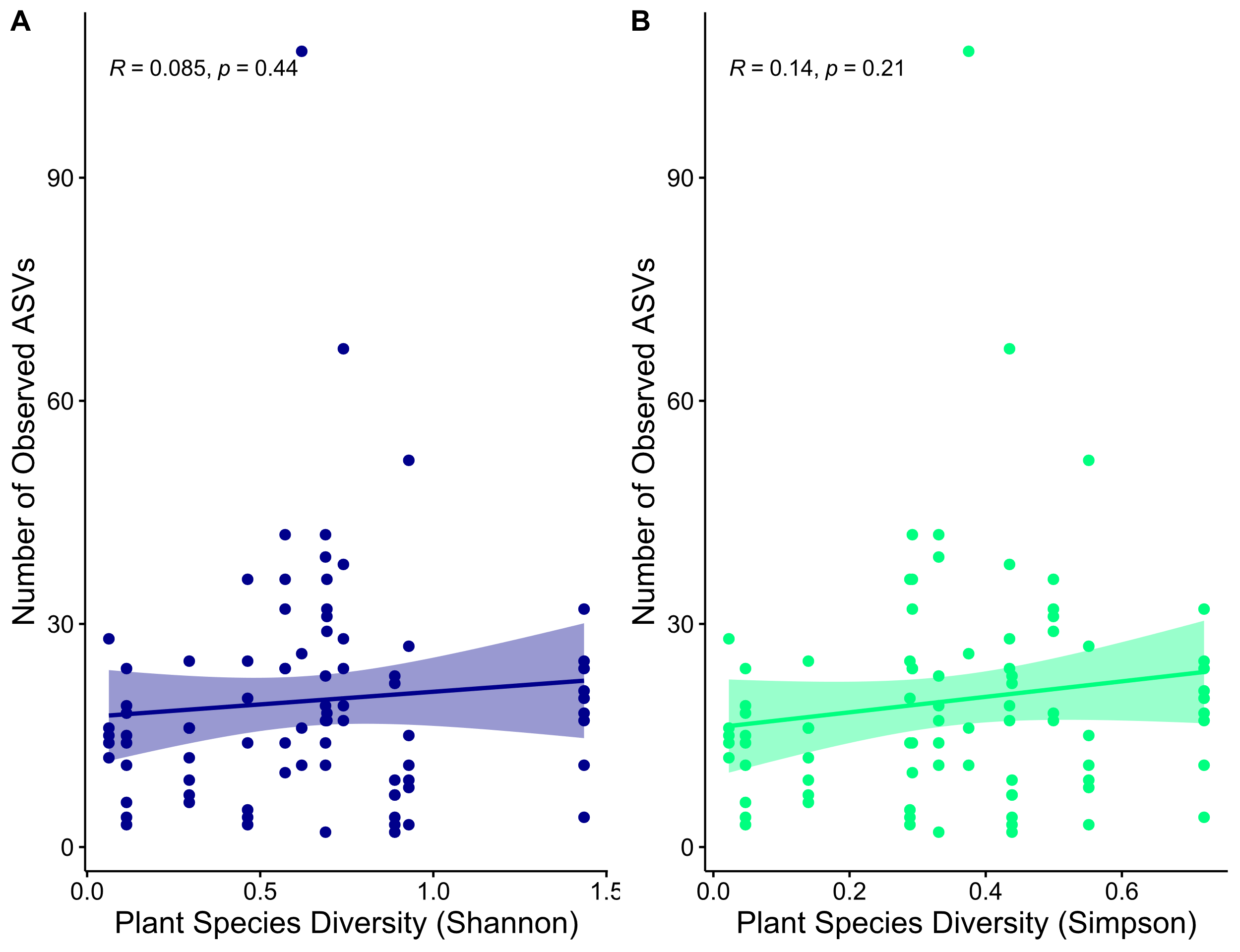


Supplementary figure S3: Correlation plots (Spearman correlation index) between seed endophytic microbiome (number of observed ASVS) and Shanon and Simpson realized plant species diversity on each plot. Plant diversity was calculated based on species biomass harvest on August 2021.
